# Natural immunity to SARS-CoV-2 and breakthrough infections in vaccinated and unvaccinated patients with cancer

**DOI:** 10.1038/s41416-022-01952-x

**Published:** 2022-08-22

**Authors:** Alessio Cortellini, Juan Aguilar-Company, Ramon Salazar, Mark Bower, Ailsa Sita-Lumsden, Andrea Plaja, Alvin J. X. Lee, Alexia Bertuzzi, Carlo Tondini, Nikolaos Diamantis, Clara Martinez-Vila, Aleix Prat, Eleanor Apthorp, Alessandra Gennari, David J. Pinato

**Affiliations:** 1 Department of Surgery & Cancer, Imperial College London, Hammersmith Hospital, London, UK; 2 Medical Oncology, Fondazione Policlinico Universitario Campus Bio-Medico, Rome, Italy; 3 Medical Oncology, Vall d’Hebron University Hospital and Institute of Oncology (VHIO), Barcelona, Spain; 4 Infectious Diseases, Vall d’Hebron University Hospital, Barcelona, Spain; 5 Department of Medical Oncology, ICO L’Hospitalet, Oncobell Program (IDIBELL), CIBERONC, Hospitalet de Llobregat, Spain; 6 Department of Oncology and National Centre for HIV Malignancy, Chelsea and Westminster Hospital, London, UK; 7 Medical Oncology, Guy’s and St Thomas’ NHS Foundation Trust (GSTT), London, UK; 8 Medical Oncology Department, B-ARGO Group, IGTP, Catalan Institute of Oncology-Badalona, Badalona, Spain; 9 Cancer Division, University College London Hospitals, London, UK; 10 Medical Oncology and Hematology Unit, Humanitas Cancer Center, IRCCS Humanitas Research Hospital, Rozzano, Milan, Italy; 11 Oncology Unit, ASST Papa Giovanni XXIII, Bergamo, Italy; 12 Medical Oncology, Barts Health NHS Trust, London, UK; 13 Fundació Althaia Manresa, Manresa, Spain; 14 Translational Genomics and Targeted Therapies in Solid Tumors, IDIBAPS, Barcelona, Spain; 15 Medical School, King’s College London, London, UK; 16 Division of Oncology, Department of Translational Medicine, University of Piemonte Orientale, Novara, Italy

**Keywords:** Oncology, Public health

## Abstract

**Background:**

Consolidated evidence suggests spontaneous immunity from SARS-CoV-2 is not durable, leading to the risk of reinfection, especially in the context of newly emerging viral strains. In patients with cancer who survive COVID-19 prevalence and severity of SARS-CoV-2 reinfections are unknown.

**Methods:**

We aimed to document natural history and outcome from SARS-CoV-2 reinfection in patients recruited to OnCovid (NCT04393974), an active European registry enrolling consecutive patients with a history of solid or haematologic malignancy diagnosed with COVID-19.

**Results:**

As of December 2021, out of 3108 eligible participants, 1806 COVID-19 survivors were subsequently followed at participating institutions. Among them, 34 reinfections (1.9%) were reported after a median time of 152 days (range: 40–620) from the first COVID-19 diagnosis, and with a median observation period from the second infection of 115 days (95% CI: 27–196). Most of the first infections were diagnosed in 2020 (27, 79.4%), while most of reinfections in 2021 (25, 73.5%). Haematological malignancies were the most frequent primary tumour (12, 35%). Compared to first infections, second infections had lower prevalence of COVID-19 symptoms (52.9% vs 91.2%, *P* = 0.0008) and required less COVID-19-specific therapy (11.8% vs 50%, *P* = 0.0013). Overall, 11 patients (32.4%) and 3 (8.8%) were fully and partially vaccinated against SARS-CoV-2 before the second infection, respectively. The 14-day case fatality rate was 11.8%, with four death events, none of which among fully vaccinated patients.

**Conclusion:**

This study shows that reinfections in COVID-19 survivors with cancer are possible and more common in patients with haematological malignancies. Reinfections carry a 11% risk of mortality, which rises to 15% among unvaccinated patients, highlighting the importance of universal vaccination of patients with cancer.

## Introduction

In the general population the seroconversion rate following Severe Acute Respiratory Syndrome Coronavirus 2 (SARS-CoV-2) infection exceeds 95%, with clear evidence of neutralising antibody response leading to protective immunity [1]. Whilst anti-SARS-CoV-2 antibodies may not be an optimal predictive correlate of protection from SARS-CoV-2, several studies have shown a decline in neutralising antibody titres over time [2, 3]. In addition, even though SARS-CoV-2 infection can elicit a potent, antigen-specific memory T-cell response in the host [4], evidence suggests that virus-specific CD4 + and CD8 + T cells may lose protective capacity over time [5]. Waning immunity from SARS-CoV-2 and the emergence of new viral strains with varying capacity to escape immunity [6] are key immunologic mechanisms explaining mounting cases of SARS-Cov-2 reinfections [7, 8]. Whilst mechanisms underlying natural and vaccinal immunity are different [9], the question of durability of protection is central to vaccinal efficacy, having led to the recommendation to offer “booster” doses in an attempt to prevent reinfection and adverse consequences from it [10].

Patients with cancer are especially vulnerable from coronavirus disease 2019 (COVID-19) [11], but similar degree of seroconversion after recovery compared to the general population has been reported from large real-world case series [12]. However, weaker immune responses to the infection, with possible implications for the risk of reinfection, are reported [13]. No reliable evidence exists as to the prevalence and severity of SARS-CoV-2 reinfections in patients with cancer. To address this gap in knowledge, we interrogated the OnCovid study and described the natural course and clinical outcome of SARS-CoV-2 breakthrough infections after prior COVID-19 in our study population.

## Methods

OnCovid (NCT04393974) is an active European registry study that, since the beginning of the pandemic, has collected data from consecutive patients with a history of solid or haematologic malignancy diagnosed with COVID-19. Patients’ observation time started from the date of first SARS-CoV-2 infection confirmation until patient’ death or loss to follow-up. Clinical information of patients who survived COVID-19 and were subsequently followed at the participating institutions according to local practice are regularly entered the registry in the context of the post-COVID-19 follow-up analysis [14–16].

In the present analysis, we evaluated the prevalence and clinical characteristics of SARS-CoV-2 breakthrough infections among COVID-19 survivors who underwent a formal clinical post-COVID-19 follow-up in the study population.

By the data lock of 22nd of December 2021, the registry included 3237 consecutive patients from 37 institutions across six countries (UK, Italy, Spain, France, Belgium and Germany), and included patients diagnosed with COVID-19 between February 27, 2020 and November 30, 2021. A list of the participating centres with eligible patients for this analysis is provided in Supplementary Table 1.

We first described baseline demographics and oncological characteristics of patients who experienced a second SARS-CoV-2 infection among the study population. The vaccination status prior the first and the second infection was also reported. COVID-19 sequelae stemming from the first SARS-CoV-2 infection were also described in patients who underwent a clinical assessment between the two infections. We compared COVID-19 symptoms and COVID-19 severity between the first and the second infections. As measures of severity, we described proportions of COVID-19 complications, receipt of COVID-19-specific therapy, hospitalisation rates and requirement for oxygen therapy. We established the all-cause case fatality rate (CFR) at 14 days following the second infection as a measure of COVID-19-related mortality, in an attempt of differentiating early (COVID-19 related) from late (cancer-related) mortality as already done in with our registry [17].

SARS-CoV-2 vaccination status prior to both infections were also reported. Patients were categorised as fully vaccinated if they had received two doses for the BNT162b2, mRNA-1273 and ChAdOx1-S vaccines at least 14 days prior to COVID-19 diagnosis or in case of infection diagnosed at least 28 days after a single dose of the Ad.26.COV2.S vaccine. Patients who received at least one vaccination, without meeting the above-mentioned criteria, were considered partially vaccinated. A detailed description of study methodology is provided in Supplementary Methods.

## Results

**Fig. 1:**
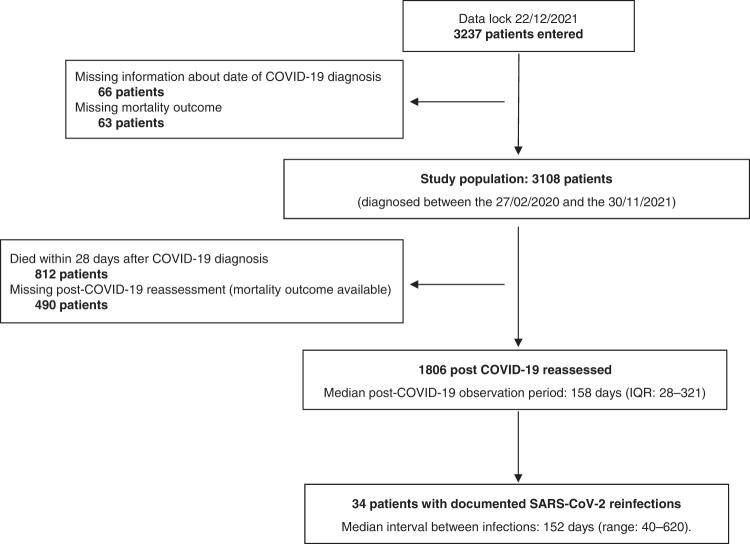
Study flowchart. IQR interquartile range.

After the exclusion of 129 patients (66 due to missing date of COVID-19 diagnosis, 63 due to missing mortality outcome), out of 3108 eligible participants, 1806 COVID-19 survivors were subsequently followed at participating institutions, with a median post-COVID-19 observation period of 158 days (interquartile range: 28–321 days). Among the 1302 excluded patients, 812 (62.4%) died within 28 days of COVID-19 diagnosis whilst post-COVID-19 information was not available for 490 (37.6%) patients. Overall, 34 reinfections (1.9%) were reported after a median time of 152 days (range: 40–620) from the first COVID-19 diagnosis (Fig. 1). Baseline demographics and oncological characteristics are reported in Supplementary Table 2. The majority of patients were female (18, 52.9%), aged ≥65 years old (20, 58.8%) and presented at least one comorbidity (26, 76.5%). The most frequent primary tumour were haematological malignancies (12, 35%), of which 4 lymphoma (4, 33.3%), 1 Hodgkin’s disease (8.3%), 2 (16.7%) multiple myeloma, 2 (16.7%) myeloid leukaemia.

No reinfected patient was fully vaccinated against SARS-CoV-2 prior to the first infection. Overall, 11 (32.4%) patients were receiving systemic anticancer therapy (SACT) at first COVID-19, in detail: 4 (36.4%) chemotherapy (either alone or in combination with other agents), 1 (9.1%) immune checkpoint inhibitors, 5 (45.4%) monoclonal antibodies/tyrosine kinase inhibitors, and 1 (9.1%) endocrine therapy. Of note, among patients on SACT at the moment of the first infection (within 4 weeks prior to COVID-19), one (9.1%) permanently discontinued the treatment, five (45.4%) resumed the treatment following a regime/dose adjustment, and four (36.4%) continued the same regimen without changes.

Among the 29 patients who underwent a clinical assessment between the two infections, 7 (25%) reported COVID-19 sequelae (6, 85.7% respiratory, 2, 28.6% others). A stage migration from non-advanced to advanced disease in-between infections was reported for only 1 (2.9%) patient.

Viral genome sequencing was not available in our database. Paired exact timings for each of the first/second infections are reported in Supplementary Table 3; most of the first infections were diagnosed in 2020 (27, 79.4%), while most of reinfections in 2021 (25, 73.5%).

**Fig. 2:**
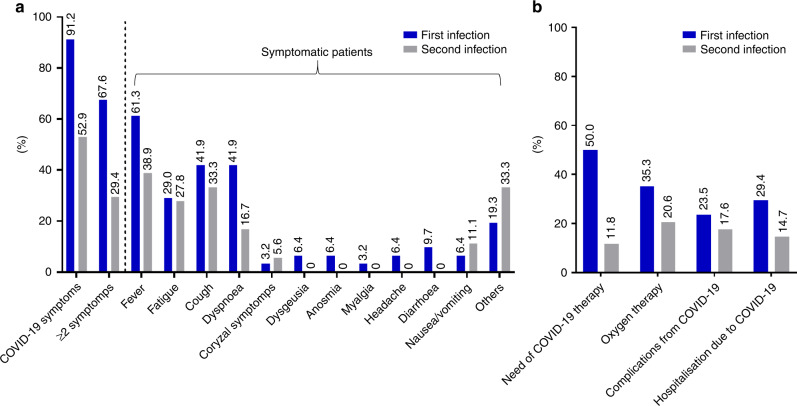
Histogram plot reporting COVID-19 symptoms and surrogates of COVID-19 severity for first and second infections. Visual summary of COVID-19 symptoms (**a**) and proxies of COVID-19 severity (**b**) prevalence across infections. The prevalence of each specific symptom is computed using the number of symptomatic patients as the denominator.

**Fig. 3:**
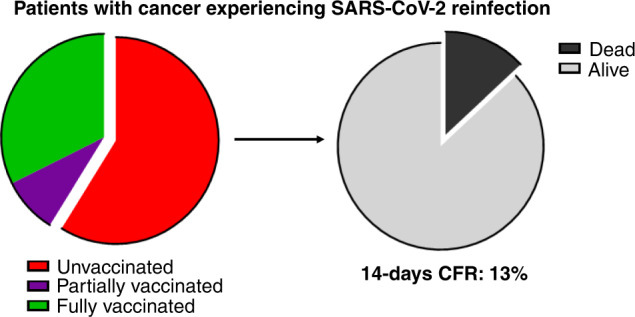
COVID-19 mortality according to vaccination status. Visual summary of the 14-day case fatality rates among patients experiencing SARS-CoV-2 reinfection.

Table 1 summarises COVID-19 symptoms and characteristics of COVID-19 severity prevalence across infections. A lower prevalence of symptoms (52.9% vs 91.2%, *P* = 0.0008) and need of COVID-19 specific therapy (11.8% vs 50%, *P* = 0.0013) was reported for the second infection as compared to the first (Fig. 2), while no difference in terms of hospitalisation (*P* = 0.2636), intensive care unit admission (*P* = 1.0000), oxygen therapy (*P* = 0.1552) and mechanical ventilation (*P* = 1.0000) requirements were found. Of note, 11 patients (32.4%) and 3 (8.8%) were fully and partially vaccinated against SARS-CoV-2 before the 2nd infection, respectively. The prevalence of COVID-19 symptoms at the second infection was slightly lower among fully vaccinated patients (63.6%, 7/11) than unvaccinated patients (70.0%, 14/2), while the need of COVID-19 therapy was slightly higher (18.2%, 2/11 vs 15.0%, 3/20). The median follow-up from the second infection was 115 days (95% CI: 27–196) and the 14-day CFR was 11.8%, with 4 death events overall, none of which occurred among fully vaccinated patients. Among 20 unvaccinated patients prior to the second infection, 3 deaths occurred within 14 days from COVID-19 diagnosis, with a 14-day CFR of 15% (Fig. 3). Among patients who died within 14 days from the second infection, most prevalent primary disease were gastrointestinal tumours [2], followed by breast cancer [1] and thoracic malignancy [1], while three patients (75%) had advanced-stage cancer.

**Table 1:** Comparison of COVID-19 symptoms and surrogates of COVID-19 severity between the first and the second infections.

	First infection	Second infection	
	*N* = 34 (%)	*N* = 34 (%)	
*Presenting COVID-19 symptoms*			
No	3 (8.8)	16 (47.1)	0.0008
Yes	31 (91.2)	18 (52.9)	
Fever	19 (61.3)	7 (38.9)	
Fatigue	9 (29)	5 (27.8)	
Cough	13 (41.9)	6 (33.3)	
Dyspnoea	13 (41.9)	3 (16.7)	
Coryzal symptoms	1 (3.2)	1 (5.6)	
Dysgeusia	2 (6.4)	–	
Anosmia	2 (6.4)	–	
Myalgia	1 (3.2)	–	
Headache	2 (6.4)	–	
Diarrhoea	3 (9.7)	–	
Nausea/vomiting	2 (6.4)	2 (11.1)	
Others	6 (19.3)	6 (33.3)	
≥2 symptoms	23 (67.6)	10 (29.4)	0.0003
*Need of COVID-19 oriented therapy*			
No	17 (50)	30 (88.2)	0.0013
Yes	17 (50)	4 (11.8)	
Chloroquine/hydroxicholoroquine	7 (41.2)	–	
Corticosteroids	8 (47.2)	3 (75)	
Antivirals	4 (23.6)	2 (50)	
Interleukin-6 inhibitors	1 (3.2)	2 (50)	
Others	2 (6.4)	2 (50)	
*Complicated COVID-19*			
No	26 (76.5)	28 (82.4)	0.5516
Yes	8 (23.5)	6 (17.6)	
Acute cardiac injury	1 (12.5)	1 (16.6)	
Acute kidney injury	1 (12.5)	2 (33.3)	
ARDS/acute respiratory failure	6 (75)	2 (33.3)	
Secondary infection	2 (25)	2 (33.3)	
Others	1 (12.5)	2 (33.3)	
*Hospitalisation*			
Not required	10 (29.4)	15 (44.1)	0.2636
Required due to COVID-19	10 (29.4)	5 (14.7)	
Pre-existing	14 (41.2)	14 (41.2)	
*Oxygen therapy requirement*			
No	21 (64.7)	27 (79.4)	0.1552
Yes	12 (35.3)	7 (20.6)	
*Intensive care unit admission*			
No	32 (94.1)	32 (94.1)	1.0000
Yes	2 (5.9)	2 (5.9)	
*Mechanical ventilation requirement*			
No	32 (94.1)	33 (97.1)	1.0000
Yes	2 (5.9)	1 (2.9)

## Discussion

This study is the first to report on SARS-CoV-2 breakthrough infections in patients with cancer who developed prior natural immunity, suggesting that ~2% of patients with cancer can experience reinfections. Although outpacing the initial <1% rate reported among the general population [18], this result should be seen in the context of the study recruitment timeframe, which covers infections contracted up to December 2021, when the reported reinfection rates for the general population ranged from 1.4 and 6.1% [19, 20].

We found a high proportion of patients with haematological malignancies amongst second infections, a finding that is aligned to the lower seroconversion rate following natural infection in this population [21] and also reflects the evidence of at least partially impaired immunogenicity of COVID-19 vaccines in patients with cancer, especially in those with haematological malignancies [22, 23].

Another factor potentially involved in defining the incidence of reinfections in patients with cancer is the deployment of periodic asymptomatic SARS-CoV-2 screening, which was largely recommended in the years 2020/2021 ahead of anticancer therapy or other diagnostic/therapeutic procedures [24, 25], although clear evidence of its clinical utility is still lacking [26–28].

In our study, SARS-Cov-2 reinfections appear to lead to milder disease manifestations, characterised by a lower symptomatic burden, reduced need for COVID-19 specific therapy mirrored by lower rates of hospitalisations, complications and oxygen therapy requirement. Whilst the reported 14-day CFR of 11.8% from reinfection is lower than ~30% CFRs for primary COVID-19 reported from most Cancer & Covid registries, including Oncovid, during the first phase of the pandemic [11] this is comparable to more modern estimates of COVID-19 mortality, where a reduction in CFR to ~14% was seen as a likely result of increased testing capacity and improved disease management [17], factors that in turn could even be linked to the lower rate of COVID-19 symptoms and therapy requirement reported during the second infection. On the other hand, the reinfected population carries an inherent selection towards negative baseline features for COVID-19 outcomes compared to primary infections reports, being haematological malignancies more represented [29].

Although this is a preliminary analysis with a limited sample size, it is important to note that no deaths were reported within 14 days from infection among fully vaccinated patients. On the other hand, the 14-days CFR for unvaccinated patients was 13%. These results, placed on the background of vaccine hesitancy, which can affect a considerable population of patients with cancer [30, 31], further highlight the importance of immunisation campaigns even in patients who gained natural immunity after a previous infection. At the same time, ~50% of reinfections were diagnosed during late 2020/early 2021, when first immunisation campaigns were still ongoing in European countries.

In our study, reinfections were diagnosed in absence of a pre-defined, prospectively planned re-testing strategy: a clear limitation that may have led to the underestimation of reinfection rates. On the other hand, patients with cancer are used to undergo regular asymptomatic SARS-CoV-2 screening, as above-mentioned [26–28]. In addition, our analysis lacked viral genomic data, an important factor to consider given the different capacities of viral strains to evade immunity. Nonetheless, our detailed reconstruction of timing of infection supports that most reinfections occurred when new variants, including the B1.351 and B.1.617.2, were at their peak of community transmission, suggesting viral immune escape to be an important determinant of the reinfection risk [32, 33], and supporting the need to monitor clinical outcomes from recurrent infections in patients with cancer [34]. Lastly, as an additional source of bias, we need to acknowledge that despite mortality outcome availability, not all COVID-19 survivors in our study population were subsequently followed at participating centres, therefore we could not reconstruct the exact prevalence of reinfections for the whole population.

Despite these limitations, this is, to our knowledge, the first study to document SARS-CoV-2 reinfections in patients with cancer. Whilst documenting comparable reinfection prevalence to the general population, our study highlights haemato-oncology patients to be at high risk of reinfection. The mortality rates in excess of 11%, occurring exclusively in unvaccinated patients experiencing a second infection, highlights the importance of adequate vaccinal coverage in patients with cancer as a measure to protect from adverse consequences from COVID-19.

## Supplementary information

Supplementary Table 1

Supplementary Table 2

Supplementary Table 3

Supplementary Methods

Conflict of interest Statement

Conflict of interest Statement

## Data Availability

Study data made available upon reasonable request.

## References

[1] Seow J, Graham C, Merrick B, Acors S, Pickering S, Steel KJA (2020). Longitudinal observation and decline of neutralizing antibody responses in the three months following SARS-CoV-2 infection in humans. Nat Microbiol.

[2] Muecksch F, Wise H, Batchelor B, Squires M, Semple E, Richardson C (2020). Longitudinal serological analysis and neutralizing antibody levels in coronavirus disease 2019 convalescent patients. J Infect Dis.

[3] Crawford KHD, Dingens AS, Eguia R, Wolf CR, Wilcox N, Logue JK (2020). Dynamics of neutralizing antibody titers in the months after severe acute respiratory syndrome coronavirus 2 infection. J Infect Dis.

[4] Rodda LB, Netland J, Shehata L, Pruner KB, Morawski PA, Thouvenel CD (2021). Functional SARS-CoV-2-specific immune memory persists after mild COVID-19. Cell..

[5] Dan JM, Mateus J, Kato Y, Hastie KM, Yu ED, Faliti CE (2021). Immunological memory to SARS-CoV-2 assessed for up to 8 months after infection. Science..

[6] Harvey WT, Carabelli AM, Jackson B, Gupta RK, Thomson EC, Harrison EM (2021). SARS-CoV-2 variants, spike mutations and immune escape. Nat Rev Microbiol.

[7] Harrington D, Kele B, Pereira S, Couto-Parada X, Riddell A, Forbes S (2021). Confirmed reinfection with severe acute respiratory syndrome coronavirus 2 (SARS-CoV-2) variant VOC-202012/01. Clin Infect Dis.

[8] Zucman N, Uhel F, Descamps D, Roux D, Ricard J-D (2021). Severe reinfection with South African severe acute respiratory syndrome coronavirus 2 (SARS-CoV-2) variant 501Y.V2. Clin Infect Dis.

[9] Gyssens IC, Netea MG (2019). Heterologous effects of vaccination and trained immunity. Clin Microbiol Infect.

[10] Cohen JI, Burbelo PD (2020). Reinfection with SARS-CoV-2: implications for vaccines. Clin Infect Dis.

[11] Desai A, Mohammed TJ, Duma N, Garassino MC, Hicks LK, Kuderer NM (2021). COVID-19 and cancer: a review of the registry-based pandemic response. JAMA Oncol.

[12] Patel M, Felip E, Sharkey R, Krengli M, Chester JD, Sita-Lumsden A (2021). 1588P SARS-CoV-2 antibody seroprevalence and safety of vaccines in cancer patients who recovered from COVID-19. Ann Oncol.

[13] Esperança-Martins M, Gonçalves L, Soares-Pinho I, Gomes A, Serrano M, Blankenhaus B (2021). Humoral immune response of SARS-CoV-2-infected patients with cancer: influencing factors and mechanisms. Oncologist.

[14] Cortellini A, Gennari A, Pommeret F, Patel G, Newsom-Davis T, Bertuzzi A (2022). COVID-19 sequelae and the host proinflammatory response: an analysis from the OnCovid registry.. JNCI: J Natl Cancer Institute.

[15] Cortellini A, Salazar R, Gennari A, Aguilar-Company J, Bower M, Bertuzzi A (2022). Persistence of long-term COVID-19 sequelae in patients with cancer: an analysis from the OnCovid registry. Eur J Cancer.

[16] Pinato DJ, Tabernero J, Bower M, Scotti L, Patel M, Colomba E (2021). Prevalence and impact of COVID-19 sequelae on treatment and survival of patients with cancer who recovered from SARS-CoV-2 infection: evidence from the OnCovid retrospective, multicentre registry study. Lancet Oncol.

[17] OnCovid Study G, Pinato DJ, Patel M, Scotti L, Colomba E, Dolly S (2022). Time-dependent COVID-19 mortality in patients with cancer: an updated analysis of the OnCovid registry. JAMA Oncol.

[18] Vitale J, Mumoli N, Clerici P, De Paschale M, Evangelista I, Cei M (2021). Assessment of SARS-CoV-2 reinfection 1 year after primary infection in a population in Lombardy, Italy. JAMA Intern Med.

[19] Flacco ME, Acuti Martellucci C, Soldato G, Carota R, Fazii P, Caponetti A, et al. Rate of reinfections after SARS-CoV-2 primary infection in the population of an Italian province: a cohort study. J Public Health (Oxf). 2021:fdab346. 10.1093/pubmed/fdab346. 10.1093/pubmed/fdab346 PMC8522392 34492110

[20] COVID-19 daily dashboard amended to include reinfections. https://www.gov.uk/government/news/covid-19-daily-dashboard-amended-to-include-reinfections.

[21] Passamonti F, Romano A, Salvini M, Merli F, Porta MGD, Bruna R (2021). COVID-19 elicits an impaired antibody response against SARS-CoV-2 in patients with haematological malignancies. Br J Haematol.

[22] Becerril-Gaitan A, Vaca-Cartagena BF, Ferrigno AS, Mesa-Chavez F, Barrientos-Gutierrez T, Tagliamento M (2022). Immunogenicity and risk of severe acute respiratory syndrome coronavirus 2 (SARS-CoV-2) infection after coronavirus disease 2019 (COVID-19) vaccination in patients with cancer: a systematic review and meta-analysis. Eur J Cancer.

[23] Fendler A, Shepherd STC, Au L, Wilkinson KA, Wu M, Byrne F (2021). Adaptive immunity and neutralizing antibodies against SARS-CoV-2 variants of concern following vaccination in patients with cancer: the CAPTURE study. Nat Cancer..

[24] NCCN Best Practices Guidance: Management of COVID-19 Infection in Patients with Cancer. https://www.nccn.org/docs/default-source/covid-19/2021-covid-infectious-disease-management.pdf?sfvrsn=63f70c30_7.

[25] Guidance on SARS-CoV-2 antigen testing for asymptomatic heathcare workers (HCW) and patients in non-surgical oncology in the UK. https://www.rcr.ac.uk/sites/default/files/guidance-covid19-testing-asymptomatic-hcw-patients-oncology.pdf.

[26] Haradaa G, Antonacio FF, Gongora AB, Behar MH, Capareli FC, Bastos DA (2020). SARS-CoV-2 testing for asymptomatic adult cancer patients before initiating systemic treatments: a systematic review. Ecancermedicalscience..

[27] Shah MA, Mayer S, Emlen F, Sholle E, Christos P, Cushing M (2020). Clinical screening for COVID-19 in asymptomatic patients with cancer. JAMA Netw Open..

[28] Meti N, Tahmasebi H, Leahey A, Boudreau A, Thawer A, Stewart J (2021). SARS-CoV-2 testing for asymptomatic patients with cancer prior and during treatment: a single centre experience. Curr Oncol..

[29] Lee LYW, Cazier JB, Starkey T, Briggs SEW, Arnold R, Bisht V (2020). COVID-19 prevalence and mortality in patients with cancer and the effect of primary tumour subtype and patient demographics: a prospective cohort study. Lancet Oncol.

[30] Nguyen M, Bain N, Grech L, Choi T, Harris S, Chau H, et al. COVID-19 vaccination rates, intent, and hesitancy in patients with solid organ and blood cancers: a multicenter study. Asia-Pacific J Clin Oncol. 2022. 10.1111/ajco.13754. 10.1111/ajco.13754 35043559

[31] Villarreal-Garza C, Vaca-Cartagena BF, Becerril-Gaitan A, Ferrigno AS, Mesa-Chavez F, Platas A (2021). Attitudes and factors associated with COVID-19 vaccine hesitancy among patients with breast cancer. JAMA Oncol.

[32] Planas D, Bruel T, Grzelak L, Guivel-Benhassine F, Staropoli I, Porrot F (2021). Sensitivity of infectious SARS-CoV-2 B.1.1.7 and B.1.351 variants to neutralizing antibodies. Nat Med.

[33] Mlcochova P, Kemp SA, Dhar MS, Papa G, Meng B, Ferreira IATM (2021). SARS-CoV-2 B.1.617.2 Delta variant replication and immune evasion. Nature..

[34] Pinato DJ, Aguilar-Company J, Ferrante D, Hanbury G, Bower M, Salazar R (2022). Outcomes of the SARS-CoV-2 omicron (B.1.1.529) variant outbreak among vaccinated and unvaccinated patients with cancer in Europe: results from the retrospective, multicentre, OnCovid registry study. Lancet Oncol.

