# Supplementary material for: Natural immunity to SARS-CoV-2 and breakthrough infections in vaccinated and unvaccinated patients with cancer
Source: Br J Cancer. 2022 Aug 22;127(10):1787–92. doi: 10.1038/s41416-022-01952-x (PMC9395853; doi:10.1038/s41416-022-01952-x)
Supplement: Supplementary file 1 — Supplementary Table 1 [file 41416_2022_1952_MOESM1_ESM.docx]

**Natural immunity to SARS-CoV-2 and breakthrough infections in vaccinated and unvaccinated patients with cancer**

Cortellini A. et al.

**Supplementary Table 1.** Patient disposition across participating centres.

| **Institution** | **Total patients** | | **Eligible** | |
| --- | --- | --- | --- | --- |
|  | **N** | **%** | **N** | **%** |
| Ospedale Antonio e Biagio e Cesare Arrigo, Alessandria (Italy) | 69 | 2.1% | - | - |
| Ospedali Riuniti di Ancona, Universitá Politecnica delle Marche (Italy) | 25 | 0.8% | - | - |
| ICO Badalona (Spain) | 62 | 1.9% | 2 | 5.9 |
| Hospital Clinic, Barcelona (Spain) | 53 | 1.6% | 2 | 5.9 |
| ICO L’Hospitalet, L’Hospitalet de Llobregat, Barcelona (Spain) | 160 | 4.9% | 4 | 11.8 |
| Vall d’Hebron University Hospital, Barcelona (Spain) | 420 | 13.0% | 4 | 11.8 |
| University of Bari 'Aldo Moro', Bari (Italy) | 5 | 0.2% | - | - |
| Ospedale Papa Giovanni XXIII, Bergamo (Italy) | 107 | 3.3% | 1 | 2.9 |
| Azienda Ospedaliera Spedali Civili, Brescia (Italy) | 26 | 0.8% | - | - |
| Fondazione Poliambulanza Istituto Ospedaliero, Brescia (Italy) | 13 | 0.4% | - | - |
| Institut Jules Bordet, Brussels (Belgium) | 30 | 0.9% | - | - |
| Velindre Cancer Centre, Cardiff (UK) | 35 | 1.1% | - | - |
| Azienda Istituti Ospitalieri di Cremona, Cremona (Italy) | 26 | 0.8% | - | - |
| Careggi University Hospital, Florence (Italy) | 14 | 0.4% | - | - |
| IRCCS AOU San Martino, Genova (Italy) | 68 | 2.1% | - | - |
| ICO Girona (Spain) | 105 | 3.2% | - | - |
| Imperial College London, London (UK) | 61 | 1.9% | - | - |
| University of L’Aquila, L’Aquila (Italy) | 34 | 1.1% | - | - |
| Santa Maria Goretti Hospital, Latina (Italy) | 12 | 0.4% | - | - |
| Chelsea and Westminster Hospital, London (UK) | 419 | 12.9% | 3 | 8.8 |
| Guy’s and St Thomas’ NHS Foundation Trust, London (UK) | 156 | 4.8% | 2 | 5.9 |
| Hospital Universitario 12 de Octubre, Madrid (Spain) | 15 | 0.5% | - | - |
| Manresa Hospital (Spain) | 46 | 1.4% | 1 | 2.9 |
| IRCCS Humanitas Research Hospital, Rozzano - Milan (Italy) | 168 | 5.2% | 2 | 5.9 |
| Istituto Europeo di Oncologia, Milano (Italy) | 15 | 0.5% | - | - |
| Istituto Tumori, Milan (Italy) | 15 | 0.5% | - | - |
| University of Munich (Germany) | 19 | 0.6% | - | - |
| Northumbria Healthcare NHS (UK) | 20 | 0.6% | - | - |
| Ospedale Maggiore della Carità, Novara (Italy) | 236 | 7.3% | 4 | 11.8 |
| Palma de Mallorca Hospital, Palma de Mallorca, (Spain) | 4 | 0.1% | - | - |
| Policlinico San Matteo, Pavia (Italy) | 99 | 3.1% | - | - |
| Azienda Ospedaliera S. Andrea, Rome (Italy) | 14 | 0.4% | - | - |
| Università Campus Bio-Medico, Rome (Italy) | 20 | 0.6% | - | - |
| Azienda Ospedaliera S Maria, Terni (Italy) | 15 | 0.5% | 1 | 2.9 |
| University College London, London (UK) | 306 | 9.5% | 7 | 20.6 |
| Barts Health NHS Trust, London (UK) | 155 | 4.8% | 1 | 2.9 |
| Institut Gustave Roussy, Villejuif (France) | 190 | 5.9% | - | - |
| **Total** | **3237** | **100.0%** | **34** | **100%** |
