# Supplementary material for: Natural immunity to SARS-CoV-2 and breakthrough infections in vaccinated and unvaccinated patients with cancer
Source: Br J Cancer. 2022 Aug 22;127(10):1787–92. doi: 10.1038/s41416-022-01952-x (PMC9395853; doi:10.1038/s41416-022-01952-x)
Supplement: Supplementary file 2 — Supplementary Table 2 [file 41416_2022_1952_MOESM2_ESM.docx]

**Natural immunity to SARS-CoV-2 and breakthrough infections in vaccinated and unvaccinated patients with cancer**

Cortellini A. et al.

**Supplementary Table 2.** Baseline demographics and oncological characteristics of patients who experienced a 2^nd^ SARS-CoV-2 infection. COPD: chronic obstructive disease; SACT: systemic anticancer therapy, Mabs: monoclonal antibodies, TKIs: tyrosine kinase inhibitors. * Within 4 weeks of COVID-19 diagnosis.

|  | **Total** |
| --- | --- |
|  | **N = 34 (%)** |
| ***Contry*** |  |
| *United Kingdom* | 13 (38.2) |
| *Spain* | 13 (38.2) |
| *Italy* | 8 (23.5) |
| ***Ethnicity*** |  |
| *White* | 28 (82.3) |
| *Black* | 1 (2.9) |
| *Asian* | 4 (11.7) |
| *Others* | 1 (2.9) |
| ***Sex*** |  |
| *Male* | 16 (47.1) |
| *Females* | 18 (52.9) |
| ***Age*** |  |
| *<65 years* | 14 (41.2) |
| *≥65 years* | 20 (58.8) |
| ***Comorbidities*** |  |
| *No* | 8 (23.5) |
| *Yes* | 26 (76.5) |
| *Cardiovascular conditions* | 20 (58.8) |
| *Dementia* | 2 (5.9) |
| *Diabetes* | 9 (26.5) |
| *COPD* | 7 (20.6) |
| *Chronic kidney disease* | 4 (11.7) |
| ***Smoking history*** |  |
| *Never smokers* | 13 (38.2) |
| *Former/current smokers* | 16 (47.1) |
| *Unknown* | 5 (14.7) |
| ***Primary Tumour*** |  |
| *Breast* | 4 (11.8) |
| *Gastrointestinal* | 5 (14.7) |
| *Gynaecological/Genito-Urinary* | 5 (14.7) |
| *Thoracic* | 8 (23.5) |
| *Haematological* | 12 (35.3) |
| *Lymphoma* | 4 (33.3) |
| *Hodgkin’s disease* | 1 (8.3) |
| *Multiple Myeloma* | 2 (16.7) |
| *Myeloid Leukaemia* | 2 (16.7) |
| *Other unspecified Leukaemia* | 2 (16.7) |
| *Reticulosarcoma* | 1 (8.3) |
| ***Tumour stage at COVID-19*** |  |
| *Non advanced* | 17 (50) |
| *Advanced* | 17 (50) |
| ***Vaccination status prior to 1st infection*** |  |
| *Unvaccinated* | 33 (97.1) |
| *Fully vaccinated* | - |
| *Partially vaccinated* | 1 (2.9) |
| ***SACT at COVID-19 diagnosis**** |  |
| *No* | 23 (67.6) |
| *Yes* | 11 (32.4) |
| *Chemotherapy (+/- combinations)* | 4 (36.4) |
| *ICIs* | 1 (9.1) |
| *Mabs/TKIs* | 5 (45.4) |
| *Endocrine therapy* | 1 (9.1) |
| *Permanent cessation after COVID-19* | 1 (9.1) |
| *Dose/regimen adjustments after COVID-19* | 5 (45.4) |
| *Resumed/continued unchanged* | 4 (36.4) |
