# Supplementary material for: Natural immunity to SARS-CoV-2 and breakthrough infections in vaccinated and unvaccinated patients with cancer
Source: Br J Cancer. 2022 Aug 22;127(10):1787–92. doi: 10.1038/s41416-022-01952-x (PMC9395853; doi:10.1038/s41416-022-01952-x)
Supplement: Supplementary file 3 — Supplementary Table 3 [file 41416_2022_1952_MOESM3_ESM.docx]

**Natural immunity to SARS-CoV-2 and breakthrough infections in vaccinated and unvaccinated patients with cancer**

Cortellini A. et al.

**Supplementary Table 3.** Infections’ timings.

|  | **First infection** | **Interval (months** | **Second infection** |
| --- | --- | --- | --- |
| 1 | March 2020 | 13 | April 2021 |
| 2 | March 2020 | 11 | February 2021 |
| 3 | March 2020 | 3 | June 2020 |
| 4 | March 2020 | 4 | July 2020 |
| 5 | March 2020 | 20 | November 2021 |
| 6 | April 2020 | 10 | February 2021 |
| 7 | April 2020 | 19 | November 2021 |
| 8 | April 2020 | 14 | June 2020 |
| 9 | April 2020 | 5 | September 2020 |
| 10 | April 2020 | 4 | August 2020 |
| 11 | April 2020 | 8 | December 2020 |
| 12 | April 2020 | 19 | November 2021 |
| 13 | April 2020 | 16 | August 2021 |
| 14 | April 2020 | 19 | November 2021 |
| 15 | April 2020 | 6 | October 2020 |
| 16 | June 2020 | 17 | November 2021 |
| 17 | September 2020 | 14 | November 2021 |
| 18 | October 2020 | 3 | January 2021 |
| 19 | October 2020 | 5 | March 2021 |
| 20 | October 2020 | 2 | December 2020 |
| 21 | October 2020 | 2 | December 2020 |
| 22 | November 2020 | 5 | May 2021 |
| 23 | December 2020 | 4 | May 2021 |
| 24 | December 2020 | 11 | November 2021 |
| 25 | December 2020 | 4 | April 2021 |
| 26 | December 2020 | 4 | April 2021 |
| 27 | December 2020 | 3 | March 2021 |
| 28 | January 2021 | 5 | June 2021 |
| 29 | January 2021 | 2 | March 2021 |
| 30 | January 2021 | 5 | June 2021 |
| 31 | January 2021 | 2 | March 2021 |
| 32 | January 2021 | 7 | August 2021 |
| 33 | February 2021 | 9 | November 2021 |
| 34 | April 2021 | 4 | August 2021 |
