# Supplementary material for: Natural immunity to SARS-CoV-2 and breakthrough infections in vaccinated and unvaccinated patients with cancer
Source: Br J Cancer. 2022 Aug 22;127(10):1787–92. doi: 10.1038/s41416-022-01952-x (PMC9395853; doi:10.1038/s41416-022-01952-x)
Supplement: Supplementary file 4 — Supplementary Methods [file 41416_2022_1952_MOESM4_ESM.docx]

**Natural immunity to SARS-CoV-2 and breakthrough infections in vaccinated and unvaccinated patients with cancer**

Cortellini A. et al.

**Supplementary Methods**

**Study design and procedures**

OnCovid (NCT04393974) is an active European registry study that, since the beginning of the pandemic, has collected consecutive patients fulfilling the following inclusion criteria: 1) age ≥18 years; 2) diagnosis of SARS-CoV-2 infection confirmed by RT-PCR of a nasopharyngeal swab; 3) history of solid or hematologic malignancy, at any time during the patients' past medical history, either active or in remission at the time of COVID-19 diagnosis. Patients with a history of non-invasive/premalignant lesions or with low malignant potential (i.e., basal cell carcinoma of the skin, non-invasive carcinoma in situ of the cervix, ductal carcinoma in situ) were excluded. For hematologic malignancies, only patients with a history of oncologic diseases with defined malignant behavior (lymphoma, leukaemia, multiple myeloma) were included.

OnCovid was granted central approval by the United Kingdom Health Research Authority (20/HRA/1608) and by the corresponding research ethics committees at each participating institution. Core study data were collated from electronic medical records into a case report form designed using the Research Electronic Data Capture software (REDCap, Vanderbilt University, Nashville, TN, USA). Multi-site access and data curation was coordinated by the Medical Statistics Unit in Novara, Italy.

The overarching subgrouping of demographics, oncological and COVID-19 related features has been consistently utilised in all the publications from our registry (Cancer Discov. 2020 Jul 31;10(10):1465–74; Eur J Cancer. 2021 Jun;150:190-202; J Immunother Cancer. 2021 Mar;9(3):e002277; Cancers. 2020 Jul 8;12(7):1841; Lancet Oncol. 2021 Nov 3;S1470-2045(21)00573-8; JAMA Oncol. 2021 Nov 24) and was made necessary by the wide heterogeneity oncological diagnoses included in the registry.

The following key variables were considered as key demographic oncological characteristics for the present analysis:

• Country (United Kingdom, Spain, Italy),

• Biological sex (male vs female),

• Age (≥65 vs < 65 years),

• Number of co-morbidities (0-1 vs ≥ 2),

• Smoking status (Never vs ever smokers),

• Primary tumour (clustered as: breast, gastro-intestinal, gynaecological/genito-urinary, thoracic, others, and haematologic),

• Tumour stage (defined as advanced vs non-advanced). In details, we defined as “advanced” stage any patient with distant metastatic disease, to differentiate them from “non-advanced” patients. Disease-specific criteria (i.e. Rai, Binet criteria etc.) were utilised as appropriate to define advanced haematological malignancies.

• Receipt of systemic anticancer therapy within 4 weeks of SARS-CoV-2 infection (yes vs no),

• The receipt of SARS-Cov-2 vaccine prior to COVID-19 (patients were categorized as fully vaccinated if they had received two doses for the BNT162b2, mRNA-1273, and ChAdOx1-S vaccines at least 14 days prior to COVID-19 diagnosis or in case of infection diagnosed at least 28 days after a single dose of the Ad.26.COV2.S vaccine. Patients who received at least one vaccination, without meeting the above-mentioned criteria, were considered partially vaccinated).

The following were considered as proxy of COVID-19 severity:

• Experience of at least one COVID-19 related symptoms including: fever, cough, fatigue, dyspnoea, anosmia, dysgeusia, coryzal symptoms, diarrhoea, headache, myalgia, nausea/vomiting, sore throat, others (yes vs no);

• COVID-19 symptoms burden (0-1 vs ≥2);

• Experience of at least one COVID-19 complications including acute respiratory failure, ARDS, kidney injury, secondary infections, sepsis, septic shock, acute cardiac injury, acute liver injury and others (yes vs no);

• Receipt of any COVID-19 oriented therapy, including antivirals, antimalarials, antibiotics, corticosteroids, interleukin-6 inhibitors and others (yes vs no);

• Hospitalization requirement (pre-existent/due to COVID-19 vs not required);

• Oxygen therapy requirement, due to COVID-19 (yes vs no);

• Intensive care unit (ICU) admission requirement (yes vs no);

• Mechanical ventilation requirement, (yes vs no);

• All-cause 14-days case fatality rate (CFR), in an attempt of differentiating early (COVID-19 related) from late (cancer-related) mortality as already done in with our registry.

The COVID-19 sequelae analysis was focused on patients who underwent a formal clinical reassessment in the oncology clinic in between the two infections at each participating institution. The clinical definitions of symptoms, clinical syndromes, complications from COVID-19 followed standardised criteria published by the World Health Organization [Available from: <file:///C:/Users/aless/Downloads/9789240025035-eng.pdf>]. These were assessed by treating physicians as per local practice and when clinically indicated, for instance during clinical consultation, symptoms review, physical examination, imaging and/or laboratory findings review. Timing of follow-up was not standardized but dictated by the discretion of treating physicians as per standard of care. COVID-19 sequelae were categorized according to the system/organ involved into: respiratory symptoms (including dyspnoea and chronic chough), and others (residual fatigue, neuro-cognitive sequelae, etc.).

Oncological and disease specific variables were collected at baseline, defined at the moment of diagnosis of SARS-CoV-2 by PCR test. Characteristics of severity, complications and therapy against COVID-19 were collected throughout the observation period until full clinical resolution of COVID-19 or patients’ mortality.

Patient observation time started from date of first PCR/SARS-CoV-2 infection confirmation until patient death or loss to follow-up. Being a retrospective, observational study, the entirety of the OnCovid cohort was followed up at intervals dictated by the routine clinical practice in each participating institutions, as deemed clinically indicated by the treating physicians. All-cause of mortality was retrieved and validated by investigators at each centre by accessing patients’ electronic medical records and death certificates.

Patients were lost to follow-up when for any reason failed to attend planned follow-up appointments scheduled by the treating clinicians. Given the pragmatic nature of this registry, based on standard of care clinical practice, we could not accurately reconstruct the reasons to explain why a proportion of patients did not attend for follow-up. To avoid incurring into bias, by mislabelling patients that were lost to follow-up as potentially deceased, we decided to exclude all patients with incomplete/missing follow up data to preserve the integrity of our results. The median follow-up was estimated with the reverse Kaplan-Meier method.

Baseline characteristics were summarized as categorical variables and reported using descriptive statistics. We tested associations between categorical variables using the Fisher exact test and the Pearson χ2 test as appropriate. A p-value of <0.05 was considered statistically significant. Analyses were performed using the MedCalc® Statistical Software version 20 (MedCalc Software Ltd, Ostend, Belgium; https://www.medcalc.org; 2021).
