# Supplementary material for: Natural immunity to SARS-CoV-2 and breakthrough infections in vaccinated and unvaccinated patients with cancer
Source: Br J Cancer. 2022 Aug 22;127(10):1787–92. doi: 10.1038/s41416-022-01952-x (PMC9395853; doi:10.1038/s41416-022-01952-x)
Supplement: Supplementary file 5 — Conflict of interest Statement [file 41416_2022_1952_MOESM5_ESM.pdf]

CONFLICT OF INTEREST STATEMENT

Natural immunity to SARS-CoV-2 and breakthrough infections in vaccinated and unvaccinated patients with cancer.

As corresponding author of the abovementioned manuscript, I declare on behalf of my co-authors the following conflict of interests:

Alessio Cortellini received consulting fees from MSD, BMS, AstraZeneca, Roche; speakers' fee from AstraZeneca, MSD, Novartis and Eisai.

Alessandra Gennari has declared consulting/advisory role for Roche, MSD, Eli Lilly, Pierre Fabre, EISAI, and Daichii Sankyo; speakers bureau for Eisai, Novartis, Eli Lilly, Roche, Teva, Gentili, Pfizer, Astra Zeneca, Celgene, and Daichii Sankyo; research funds: EISAI, Eli Lilly, and Roche.

David J Pinato received lecture fees from ViiV Healthcare, Bayer Healthcare, BMS, Roche, EISAI, Falk Foundation, travel expenses from BMS and Bayer Healthcare; consulting fees for Mina Therapeutics, EISAI, Roche, DaVolterra and Astra Zeneca; research funding (to institution) from MSD and BMS.

All remaining authors have declared no conflicts of interest.

London, May 4th, 2022

Alessio Cortellini
